# Supplementary material for: Viral zoonoses assessment in invasive rodent species from São Tomé and Príncipe
Source: PLoS One. 2026 Feb 2;21(2):e0341657. doi: 10.1371/journal.pone.0341657 (PMC12863471; doi:10.1371/journal.pone.0341657)
Supplement: S1 Table — (PDF) [file pone.0341657.s001.pdf]

S1 Table. Species, sex, and body measurements of the individuals sampled in the different habitats: cacao (A), village (B), forest (C) and palm (D).

(A) Cacao

Rattus rattus						*Rattus norvegicus*				
**Body (cm)**	**Tail (cm)**	**Foot (mm)**	**Weight (g)**	**Sex**		**Body (cm)**	**Tail (cm)**	**Foot (mm)**	**Weight (g)**	**Sex**
19.0	20.0	23.5	156	M						
21.5	22.4	25.1	250							
19.9	19.9	25.6	179							
20.2	22.4	26.1	233							
21.1	21.3	25.9	176							
17.7	19.2	25.0	133							
19.6	20.2	26.1	206							
10.0	16.0	30.0	80.0							
**18.6**	**20.2**	**25.9**	**176.6**	**Average**						
19.3	20.2	22.3	169	F						
18.6	20.4	25.0	149							
14.6	16.5	23.5	89							
18.1	19.7	21.1	145							
16.5	17.0	24.0	98							
20.0	20.9	25.2	167							
15.9	20.5	22.2	107							
19.3	20.6	24.6	129							
10.6	12.9	30.0	60							
15.5	19.5	35.0	120			21.6	16.4	26.6	206	F
**16.8**	**18.8**	**25.3**	**123.3**	**Average**						

(B) Village

Rattus rattus						*Rattus norvegicus*				
**Body (cm)**	**Tail (cm)**	**Foot (mm)**	**Weight (g)**	**Sex**		**Body (cm)**	**Tail (cm)**	**Foot (mm)**	**Weight (g)**	**Sex**
19.0	16.6	24.5	140							
19.0	19.4	24.4	130							
18.5	20.4	24.5	132							
19.1	21.4	24.9	128							
18.4	20.8	23.8	141							
20.3	22.9	26.1	156	F						
18.3	21.2	23.4	112							
17.1	20.9	24.1	121			20.0	15.4	26.6	189	
17.8	20.4	23.3	125			22.6	17.3	29.5	222	F
18.3	21.0	23.1	115			19.1	14.9	25.8	178	
**18.6**	**20.5**	**24.2**	**130.0**	**Average**		**20.6**	**15.9**	**27.3**	**196.3**	**Average**
19.0	20.1	24.0	176			22.1	18.2	27.7	136	
19.1	20.3	25.9	168			23.1	18.2	27.5	313	
18.8	19.1	24.5	172	M		17.1	13.4	26.1	142	M
16.2	19.0	23.6	100			20.6	16.1	27.2	173	
19.2	21.0	23.7	130			21.4	16.6	28.5	201	
22.4	23.0	24.4	175			16.9	12.5	25.6	93	
**19.1**	**20.4**	**24.4**	**153.5**	**Average**		**20.2**	**15.8**	**27.1**	**176.3**	**Average**

(C) Forest

Rattus rattus						*Rattus norvegicus*				
**Body (cm)**	**Tail (cm)**	**Foot (mm)**	**Weight (g)**	**Sex**		**Body (cm)**	**Tail (cm)**	**Foot (mm)**	**Weight (g)**	**Sex**
20.7	20.9	26.4	204	M						
21.3	21.5	25.1	205							
21.2	22.0	26.4	222							
20.6	21.2	25.3	196							
18.4	16.0	27.2	157							
19.6	20.0	26.1	151							
14.7	17.0	24.8	91							
19.1	21.0	24.8	192							
18.0	20.2	27.2	144							
19.7	21.6	25.5	191			21.5	21.4	26.6	195	M
**19.3**	**20.1**	**25.9**	**175.3**	**Average**						
19.0	20.4	25.0	180	F						
20.6	23.5	26.0	195							
18.1	21.0	24.2	161							
19.4	21.3	25.0	176							
19.5	23.9	25.9	199							
**19.3**	**22.0**	**25.2**	**182.2**	**Average**						

(D) Palm

Rattus rattus						*Rattus norvegicus*				
**Body (cm)**	**Tail (cm)**	**Foot (mm)**	**Weight (g)**	**Sex**		**Body (cm)**	**Tail (cm)**	**Foot (mm)**	**Weight (g)**	**Sex**
19.6	20.5	23.9	167	F						
19.4	20.2	23.0	182							
17.5	17.7	23.4	106							
19.1	22.3	26.1	181							
19.0	21.0	24.5	141							
18.7	19.2	23.9	149							
20.0	21.5	24.4	175							
20.0	23.0	27.0	220			19.5	17.5	24.7	146	F
18.5	21.0	25.0	240			20.5	16.3	25.8	219	
14.0	18.0	22.0	160			21.0	18.3	22.5	174	
**19.1**	**20.7**	**24.6**	**173.4**	**Average**		**20.3**	**17.4**	**24.3**	**179.7**	**Average**
17.4	19.5	26.3	114	M						
20.7	21.0	25.1	191							
20.0	22.5	26.8	200							
21.3	21.6	25.1	195							
17.0	19.0	25.9	113							
18.5	20.6	27.4	147							
17.0	17.4	24.1	101							
20.0	21.5	-	250							
17.0	19.5	24.0	160							
19.0	23.6	-	200							
15.0	18.5	25.0	140							
**18.4**	**20.4**	**25.5**	**164.6**	**Average**						
