# Supplementary material for: Viral zoonoses assessment in invasive rodent species from São Tomé and Príncipe
Source: PLoS One. 2026 Feb 2;21(2):e0341657. doi: 10.1371/journal.pone.0341657 (PMC12863471; doi:10.1371/journal.pone.0341657)
Supplement: Attachment — Submitted filename: PONE-D-25-32689_reviewer1.pdf [file pone.0341657.s002.pdf]

PLOS ONE

Viral zoonoses assessment in invasive rodent species from São Tomé and Príncipe

--Manuscript Draft--

**Manuscript Number:**	PONE-D-25-32689
**Article Type:**	Research Article
**Full Title:**	Viral zoonoses assessment in invasive rodent species from São Tomé and Príncipe
**Short Title:**	Screening of zoonotic viruses in rodents from São Tomé and Príncipe
**Corresponding Author:**	Joana Abrantes, PhD<br>CIBIO: Universidade do Porto Centro de Investigacao em Biodiversidade e Recursos Geneticos<br>Vairão, PORTUGAL
**Keywords:**	Gulf of Guinea; introduced species; black rat; brown rat; rodent-borne viruses; public health
**Abstract:**	Zoonoses are diseases transmitted from animals to humans, highlighting the inseparable link between animal and human health. They are responsible for approximately 2.4 billion cases of illness and 2.2 million deaths annually, posing a significant challenge to public health and food security. Transmission of infectious agents from animals to humans occurs through direct contact, ingestion, inhalation, or inoculation of the infectious agent. Finding practical ways to monitor the presence and/or abundance of zoonotic pathogens is important to estimate the risk of spillover to humans. Since rodents are present almost everywhere, live in proximity with humans and host several zoonotic viruses, we conducted a screening in different tissue samples of black rats (Rattus rattus) and brown rats (Rattus norvegicus) collected in São Tomé and Príncipe in 2021 and 2022 for the presence of five zoonotic families of viruses, including Arenaviridae, Coronaviridae, Flaviviridae, Hantaviridae, and Poxviridae. Although we found no evidence of the presence of these viral taxa among the rodent samples tested, our study does not exclude their presence in São Tomé and Príncipe. Continued monitoring of these and other zoonotic viruses is advisable to prevent or mitigate the emergence of viral diseases that are often fatal to humans.
**Order of Authors:**	Tereza Almeida
	Maria Carolina Matos
	Daniel A Velarde-Garcéz
	Vanessa A Mata
	Marquinha Martins
	Conceição Neves
	Pedro Beja
	Ana M Lopes
	Joana Abrantes, PhD
**Additional Information:**	
**Question**	**Response**
**Financial Disclosure**<br><br>Enter a financial disclosure statement that describes the sources of funding for the work included in this submission. Review the submission guidelines for detailed requirements. View published research articles from *PLOS ONE* for specific	Yes

examples.<br><br>This statement is required for submission and **will appear in the published article** if the submission is accepted. Please make sure it is accurate.<br><br>**Funded studies**<br>Enter a statement with the following details:<br>• Initials of the authors who received each award<br>• Grant numbers awarded to each author<br>• The full name of each funder<br>• URL of each funder website<br>• Did the sponsors or funders play any role in the study design, data collection and analysis, decision to publish, or preparation of the manuscript?<br><br>Did you receive funding for this work?	
Please add funding details.<br>as follow-up to "**Financial Disclosure**<br><br>Enter a financial disclosure statement that describes the sources of funding for the work included in this submission. Review the submission guidelines for detailed requirements. View published research articles from *PLOS ONE* for specific examples.<br><br>This statement is required for submission and **will appear in the published article** if the submission is accepted. Please make sure it is accurate.<br><br>**Funded studies**<br>Enter a statement with the following details:<br>• Initials of the authors who received each award<br>• Grant numbers awarded to each author<br>• The full name of each funder<br>• URL of each funder website<br>• Did the sponsors or funders play any role in the study design, data collection and analysis, decision to publish, or preparation of the manuscript?	This study was done within the framework of the partnership agreement between BirdLife International and BIOPOLIS Association that promotes informed, evidence-based biodiversity conservation action in São Tomé and Príncipe, and is funded by the European Union through the "Landscape Management in São Tomé and Príncipe" project (contract number ENV/2020/420-182). The work was also supported by TROPIBIO NORTE-01-0145-FEDER-000046, funded by Norte Portugal Regional Operational Programme (NORTE2020), under the PORTUGAL 2020 Partnership Agreement, through the European Regional Development Fund (ERDF).<br>Fundação para a Ciência e a Tecnologia (FCT) funded the individual research contracts of VAM and AML (CEECIND/02547/2020, https://doi.org/10.54499/2020.02547.CEECIND/CP1601/CP1649/CT0004; CEECIND/01388/2017, https://doi.org/10.54499/CEECIND/01388/2017/CP1423/CT0007). MCM and DAVG were supported by PhD scholarships funded by FCT (2023.02359.BD and 2023.02833.BD, respectively).<br>The funders played no role in the study design, data collection and analysis, decision to publish, or preparation of the manuscript.

Powered by Editorial Manager® and ProduXion Manager® from Aries Systems Corporation

Did you receive funding for this work?"	
Please select the country of your main research funder (please select carefully as in some cases this is used in fee calculation).<br>as follow-up to "**Financial Disclosure**<br><br>Enter a financial disclosure statement that describes the sources of funding for the work included in this submission. Review the submission guidelines for detailed requirements. View published research articles from *PLOS ONE* for specific examples.<br><br>This statement is required for submission and **will appear in the published article** if the submission is accepted. Please make sure it is accurate.<br><br>**Funded studies**<br>Enter a statement with the following details:<br>• Initials of the authors who received each award<br>• Grant numbers awarded to each author<br>• The full name of each funder<br>• URL of each funder website<br>• Did the sponsors or funders play any role in the study design, data collection and analysis, decision to publish, or preparation of the manuscript?<br><br>Did you receive funding for this work?"	PORTUGAL - PT
**Competing Interests**<br><br>Use the instructions below to enter a competing interest statement for this submission. On behalf of all authors, disclose any competing interests that could be perceived to bias this work—acknowledging all financial support and any other relevant financial or non-financial competing interests.	The authors have declared that no competing interests exist.

<table>
<tr>
<td>This statement is required for submission and will appear in the published article if the submission is accepted. Please make sure it is accurate and that any funding sources listed in your Funding Information later in the submission form are also declared in your Financial Disclosure statement.

View published research articles from PLOS ONE for specific examples.

NO authors have competing interests

Enter: The authors have declared that no competing interests exist.

Authors with competing interests

Enter competing interest details beginning with this statement:

I have read the journal's policy and the authors of this manuscript have the following competing interests: [insert competing interests here]

* typeset</td>
<td></td>
</tr>
<tr>
<td>Ethics Statement

Enter an ethics statement for this submission. This statement is required if the study involved:

• Human participants
• Human specimens or tissue
• Vertebrate animals or cephalopods
• Vertebrate embryos or tissues
• Field research

Write "N/A" if the submission does not require an ethics statement.

General guidance is provided below. Consult the submission guidelines for detailed instructions. Make sure that all information entered here is included in the Methods section of the manuscript.</td>
<td>Animals were anesthetized with isoflurane and then euthanized by cervical dislocation; all efforts were made to minimize suffering.
Sampling was approved by “Direção das Florestas e da Biodiversidade” of São Tomé and Príncipe, and shipping was certified by “Centro de Investigação Agronómica e Tecnológica de São Tomé e Príncipe (CIAT-STP)”.
The protocol was approved by the Animal Welfare and Ethics Review Body of BIOPOLIS-CIBIO-InBIO (ORBEA-BIOPOLIS-CIBIO).</td>
</tr>
</table>

Powered by Editorial Manager® and ProduXion Manager® from Aries Systems Corporation

**Format for specific study types**<br><br>**Human Subject Research (involving human participants and/or tissue)**<br>• Give the name of the institutional review board or ethics committee that approved the study<br>• Include the approval number and/or a statement indicating approval of this research<br>• Indicate the form of consent obtained (written/oral) or the reason that consent was not obtained (e.g. the data were analyzed anonymously)<br><br>**Animal Research (involving vertebrate animals, embryos or tissues)**<br>• Provide the name of the Institutional Animal Care and Use Committee (IACUC) or other relevant ethics board that reviewed the study protocol, and indicate whether they approved this research or granted a formal waiver of ethical approval<br>• Include an approval number if one was obtained<br>• If the study involved *non-human primates*, add *additional details* about animal welfare and steps taken to ameliorate suffering<br>• If anesthesia, euthanasia, or any kind of animal sacrifice is part of the study, include briefly which substances and/or methods were applied<br><br>**Field Research**<br><br>Include the following details if this study involves the collection of plant, animal, or other materials from a natural setting:<br>• Field permit number<br>• Name of the institution or relevant body that granted permission	
**Data Availability**<br><br>Authors are required to make all data underlying the findings described fully available, without restriction, and from the time of publication. PLOS allows rare exceptions to address legal and ethical concerns. See the PLOS Data Policy and FAQ for detailed information.	Yes - all data are fully available without restriction

A Data Availability Statement describing where the data can be found is required at submission. Your answers to this question constitute the Data Availability Statement and **will be published in the article**, if accepted.<br><br>**Important:** Stating 'data available on request from the author' is not sufficient. If your data are only available upon request, select 'No' for the first question and explain your exceptional situation in the text box.<br><br>Do the authors confirm that all data underlying the findings described in their manuscript are fully available without restriction?	
**Describe where the data may be found in full sentences. If you are copying our sample text, replace any instances of XXX with the appropriate details.**<br><br>• If the data are **held or will be held in a public repository**, include URLs, accession numbers or DOIs. If this information will only be available after acceptance, indicate this by ticking the box below. For example: *All XXX files are available from the XXX database (accession number(s) XXX, XXX.*).<br>• If the data are all contained **within the manuscript and/or Supporting Information files**, enter the following: *All relevant data are within the manuscript and its Supporting Information files.*<br>• If neither of these applies but you are able to provide **details of access elsewhere**, with or without limitations, please do so. For example:<br><br>*Data cannot be shared publicly because of [XXX]. Data are available from the XXX Institutional Data Access / Ethics Committee (contact via XXX) for researchers who meet the criteria for access to confidential data.*<br><br>*The data underlying the results presented in the study are available*	n.a.

from (include the name of the third party and contact information or URL).<br>• This text is appropriate if the data are owned by a third party and authors do not have permission to share the data.<br><br>* typeset	
Additional data availability information:	

Powered by Editorial Manager® and ProduXion Manager® from Aries Systems Corporation

Manuscript

Click here to access/download;Manuscript;Almeida et al_Manuscript_PLoS One.docx

Title

Viral zoonoses assessment in invasive rodent species from São Tomé and Príncipe

Authors

Tereza Almeida[1,2], Maria Carolina Matos[1,2], Daniel A. Velarde-Garcéz[1,2], Vanessa A. Mata[1,2], Marquinha Martins[3], Conceição Neves[3], Pedro Beja[1,2,4], Ana M. Lopes[1,2,5], Joana Abrantes[1,2,6]*

Affiliations

[1]CIBIO, Centro de Investigação em Biodiversidade e Recursos Genéticos, InBIO Laboratório Associado, Campus de Vairão, Universidade do Porto, 4485-661 Vairão, Portugal

[2]BIOPOLIS Program in Genomics, Biodiversity and Land Planning, CIBIO, Campus de Vairão, 4485-661 Vairão, Portugal

[3]Birdlife International, São Tomé and Príncipe Office, Bairro do Hospital, Nº 2929, Água-Grande, São Tomé, São Tomé e Príncipe

[4]CIBIO, Centro de Investigação em Biodiversidade e Recursos Genéticos, InBIO Laboratório Associado, Instituto de Agronomia, Universidade de Lisboa, Lisboa, Portugal

[5]UMIB - Unit for Multidisciplinary Research in Biomedicine, ICBAS - School of Medicine and Biomedical Sciences, University of Porto, Porto, Portugal

[6]Departamento de Biologia, Faculdade de Ciências, Universidade do Porto, 4099-002 Porto, Portugal

***Corresponding author:** Joana Abrantes, jabrantes@cibio.up.pt

Abstract

Zoonoses are diseases transmitted from animals to humans, highlighting the inseparable link between animal and human health. They are responsible for approximately 2.4 billion cases of illness and 2.2 million deaths annually, posing a significant challenge to public health and food security. Transmission of infectious agents from animals to humans occurs through direct contact, ingestion, inhalation, or inoculation of the infectious agent. Finding practical ways to monitor the presence and/or abundance of zoonotic pathogens is important to estimate the risk of spillover to humans. Since rodents are present almost everywhere, live in proximity with humans and host several zoonotic viruses, we conducted a screening in different tissue samples of black rats (*Rattus rattus*) and brown rats (*Rattus norvegicus)* collected in São Tomé and Príncipe in 2021 and 2022 for the presence of five zoonotic families of viruses, including *Arenaviridae*, *Coronaviridae*, *Flaviviridae*, *Hantaviridae*, and *Poxviridae*. Although we found no evidence of the presence of these viral taxa among the rodent samples tested, our study does not exclude their presence in São Tomé and Príncipe. Continued monitoring of these and other zoonotic viruses is advisable to prevent or mitigate the emergence of viral diseases that are often fatal to humans.

Introduction

The archipelago of São Tomé and Príncipe is located on the equator in the Gulf of Guinea in West Central Africa. It includes two main islands, São Tomé and Príncipe, and several islets. The archipelago has a relatively small area of around 1000 km2 and ca. 250 km off the mainland. São Tomé and Príncipe have a unique biological diversity, with a high number of endemic species of birds, amphibians, and plants, as a result of its orography, climate and geography [1].

Discovery and colonization of the archipelago in the 1470s led to the introduction of several exotic species, such as wild pig (*Sus scrofa*), mona monkey (*Cercopithecus mona*), African civet (*Civettictis civetta*), weasel (*Mustela nivalis*), house mouse (*Mus musculus*), black rat

(*Rattus rattus*) and brown rat (*Rattus norvegicus*). Other domestic and feral mammals, such as dogs, cats, pigs, goats, cows, horses, and others, have recently colonized the archipelago. Thus, all non-flying mammals present nowadays in the archipelago of São Tomé and Príncipe, except the endemic shrews *Crocidura thomensis* and *Crocidura fingui*, were introduced [1,2].

The introduction of invasive species had a detrimental impact on the native flora and fauna of São Tomé and Príncipe, being associated with habitat degradation and loss, competition for food and space, and predation. Indeed, the predation of adults, juveniles and nests of some critically endangered species by rats, monkeys, civets, and weasels has been highlighted by BirdLife International [3]. Rats and civets have successfully colonized primary forest areas, including river edges [3], and exert a significant impact by consuming eggs and preying on juveniles. Additionally, introduced rodents can serve as reservoirs for infectious diseases such as leptospirosis, plague, toxoplasmosis, hantavirus and arenavirus infections, amplifying their impact [4].

The black rat (*Rattus rattus*) is in the top 100 worst invasive species globally due to the negative impact on the ecosystems [5]. Numerous studies detailed their significant impact on natural ecosystems, especially on islands [3,6–8]. Furthermore, they have wide-ranging implications for human activities and health, including agriculture and infrastructure damage, spread of diseases, contamination of food supplies, and increased public health costs [4,9–11]. By living in close contact with humans and domestic animals, having an almost worldwide distribution (except Antarctica and some islands) [12], they are important reservoirs of zoonotic viruses [11,13–17]. Rodents are primary mammalian reservoirs for zoonotic pathogens due to their high pathogen diversity, causing around 90 diseases, including >30 viral zoonoses alongside bacterial, helminthic, protozoan, and fungal infections [9,10].

While humans host many viruses without clinical signs, several rodent-borne viruses can cause severe, and often fatal, diseases in humans, e.g., lymphocytic choriomeningitis virus (LCMV), Lassa virus (LASV), severe acute respiratory syndrome coronavirus (SARS-CoV), Middle East respiratory syndrome coronavirus (MERS-CoV), tick-borne encephalitis virus (TBEV), dengue virus, Hantaan virus (HTNV), Seoul virus (SEOV), Puumala virus, monkeypox (MPXV) and

cowpox (CPXV) viruses, among others [11,13–17]. Humans can be infected either directly through rodent biting, contact with water, food, and surfaces contaminated with infected urine and/or feces, inhalation of aerosols, or indirectly via arthropod vectors [18].

This study aimed to detect and assess the prevalence of zoonotic viruses in invasive black and brown rats from the archipelago of São Tomé and Príncipe as human and non-human communities are more vulnerable to diseases introduced by invasive species. This vulnerability stems from ecological, biological, and social factors [19]. We focused on selected viral taxa with zoonotic potential previously detected in rodents from other African countries, namely *Poxviridae*, *Arenaviridae*, *Coronaviridae*, *Flaviviridae*, and *Hantaviridae [20–25]*. *Arenaviridae* and *Hantaviridae*, which include Lassa fever and hantavirus pulmonary syndrome-causing viruses, pose serious public health risks [e.g. 22,25]. *Coronaviridae*, including SARS-CoV-2, *Flaviviridae*, such as yellow fever and Zika viruses, and *Poxviridae* like cowpox, have also been reported in African rodents, highlighting their role in zoonotic transmission [20,21,23,24].

Materials and methods

1. Specimen collection

Sampling was carried out in two field trips: one pilot trip and one extended field season. The pilot trip took place in December 2021, during which several trapping techniques were explored in cocoa plantations, palm plantations, and forests. The second field trip occurred from May to September 2022 across 12 localities evenly spread among the following habitats: forests, cocoa and palm plantations, and villages. Rodents were captured using Tomahawk-like traps baited with oat cookies and peanut butter. Sampling in each locality lasted 4 days, in a 1-hectare plot containing 36 traps, each spaced 20 meters apart. Traps were set up on the morning of the first day and checked daily after dawn and before dusk. The species, sex, and body measurements of each captured animal were recorded. Animals were anesthetized with isoflurane and then euthanized by cervical dislocation; all efforts were made to minimize suffering. Liver, spleen and feces samples were collected and stored in RNA*later*® (Thermo Fisher Scientific) at room

temperature until the end of the day, after which they were transferred to a freezer. Samples were shipped to the laboratories of BIOPOLIS-CIBIO-InBIO, Vairão, Portugal. Collection and shipment of the samples were done according to local legislation. Sampling was approved by "Direção das Florestas e da Biodiversidade" of São Tomé and Príncipe, and shipping was certified by "Centro de Investigação Agronómica e Tecnológica de São Tomé e Príncipe (CIAT-STP)". The protocol was approved by the Animal Welfare and Ethics Review Body of BIOPOLIS-CIBIO-InBIO (ORBEA-BIOPOLIS-CIBIO).

2. DNA and RNA extraction

DNA and RNA were extracted from 10-30 mg of liver (n=81), spleen (n=81) and fecal (n=80) samples of *R. rattus* (n=71) and *R. norvegicus* (n=15) using the EasySpin Genomic DNA Tissue Kit SP-DT-250 (Citomed) and the GeneJET RNA Purification kit (Thermo Fisher Scientific), respectively, according to the manufacturer's protocol. cDNA was synthesized with the NZY first-strand cDNA synthesis kit (Nzytech), according to the provided protocol.

3. DNA viruses screening

Detection of poxviruses with zoonotic potential was performed by conventional PCR using pan-pox universal primers available in the literature (Table 1). Reactions were carried out with 1 μL of DNA in a final volume of 10 μL containing 5 μL of Phusion Flash High-Fidelity PCR Master Mix (Thermo Fisher Scientific) and 2 pmol of each oligonucleotide. Conditions for PCR amplification were: 98 ºC for 10 sec, 45 cycles of 98 ºC for 1 sec, 48ºC for 5 sec and 72 ºC for 4 sec, and a final extension at 72 ºC for 1 min. A myxoma virus (*Leporipoxvirus*) positive sample available at CIBIO/UP was included as a positive control.

Table 1. List of primers and PCR conditions used for detection of DNA viruses.

**Virus**	**Primer**	**Sequence 5'-3'**	**Amplicon**	**Reference**
Poxviridae	PanPox	ACACCAAAAACTCATATAACTTCT	220	[26]

	PanPox	CCTATTTTACTCCTTAGTAAATGAT		

4. RNA viruses screening

Detection of RNA viruses was performed by conventional RT-PCR using pan/universal primers available in the literature (Table 2). Reactions were carried out with 1 µL of cDNA in a final volume of 10 µL containing 5 µL of Phusion Flash High-Fidelity PCR Master Mix (Thermo Fisher Scientific) or Supreme NZYTaq II 2x Green Master Mix (Nzytech) and 2 pmol of each oligonucleotide. For each virus family, a positive sample was included as control of the reaction: for *Coronaviridae*, SARS-CoV-2 (kindly provided by Susana Lopes, CIBIO/UP); for *Flaviviridae*, Bagaza virus (kindly provided by João Queirós, CIBIO/UP); for *Arenaviridae*, lymphocytic choriomeningitis v[illegible] (pLCMV_Pol-I-L-ARM; provided by the University of Basel, Department of Biomedicine, as participant of the European Virus Archive); for *Hantaviridae*, Hantaan virus (HTNV 78-118; provided by the Biomedical Research Center of the Slovak Academy of Sciences, as participant of the European Virus Archive).

For coronaviruses, conditions for PCR amplification were: 98 ºC for 5 sec, 40 cycles of 98 ºC for 1 sec, 51 ºC for 5 sec and 72 ºC for 2 sec, and a final extension at 72 ºC for 1 min. The PCR product was used as a template for a second PCR to improve virus detection, using the same conditions.

For flaviviruses, conditions for PCR amplification were: 95 ºC for 5 min, 35 cycles of 94 ºC for 30 sec, 57 ºC for 25 sec and 72 ºC for 5 sec, and a final extension at 72 ºC for 10 min.

For the arenavirus detection, conditions for PCR amplification were: 98 ºC for 10 sec, 30 cycles of 98 ºC for 1 sec, 52 ºC for 5 sec and 72 ºC for 7 sec, and a final extension at 72 ºC for 1 min.

For the hantavirus detection, a nested PCR was performed that targets a conserved region of the L gene [25]. The first PCR conditions: 98 ºC for 10 sec, 7 cycles of 98 ºC for 1 sec, 50 ºC for 5 sec and 72 ºC for 15 sec, 35 cycles of 98 ºC for 1 sec, 55 ºC for 5 sec and 72 ºC for 15 sec and a final extension at 72 ºC for 1 min. The second PCR conditions: 98 ºC for 10 sec, 40 cycles of 98 ºC for 1 sec, 60ºC for 5 sec and 72 ºC for 15 sec, and a final extension at 72 ºC for 1 min.

Table 2. List of primers and PCR conditions used for detection of RNA viruses.

Virus family	Primer ID	Sequence 5'-3'	Amplicon size (bp)	Reference
Arenaviridae	LVL3359A-	AGAATTAGTGAAAGGGAGAGCAATTC	394	[27]
	LVL3359D-	AGAATCAGTGAAAGGGAAAGCAATTC		
	LVL3359G-	AGAATTAGTGAAAGGGAGAGTAACTC		
	LVL3754A-	CACATCATTGGTCCCCATTTACTATGATC		
	LVL3754D-	CACATCATTGGTCCCCATTTACTGTGATC		
Coronaviridae	PanCoV-[illegible]	TGATGATGSNGTTGTNTGYTAYAA	154	[28]
	PanCoV-[illegible]	GCATWGTRTGYTGNGARCARAATTC		
Flaviviridae	PFlav-F	TACAACATGATGGGAAAGAGAGAGAARAA	270	[29]
	PFlav-R	GTGTCCCAKCCRGCTGTGTCATC		
Hantaviridae	HAN-L F1	ATGTAYGTBAGTGCWGATGC	412	[25]
	HAN-L-R1	AACCADTCWGTYCCRTCATC		
	HAN-L- F2	TGCWGATGCHACIAARTGGTC		
	HAN-L-R2	GCRTCRTCWGARTGRTGDGCAA		

Results and Discussion

Rodents exhibit remarkable adaptability and thrive in environments closely intertwined with humans and anthropogenic activities, fostering the emergence of rodent-borne zoonotic viruses [19]. Monitoring of rodents has been proposed as an effective tool for surveilling zoonotic diseases, since they host a large plethora of viruses and play a key role in the transmission of infectious agents. In this study, we collected a total of 240 samples from 72 black rats (*R. rattus*) and 14 brown rats (*R. norvegicus*). Samples were collected in different habitats, broadly following an anthropogenic gradient, from villages to palm and cocoa plantations, and to forests, covering a wide spectrum of contact intensity between rats and humans. The samples included liver (n=81), spleen (n=80), and feces (n[illegible]). with an average of 20 samples (range

15-24) per tissue per habitat. All samples were screened using conventional PCR and RT-PCR to detect the presence of DNA or RNA from viral taxa with zoonotic potential. Selection of the taxa was based on their previous identification in rodents from other African countries, specifically *Poxviridae*, *Coronaviridae*, *Flaviviridae*, *Arenaviridae*, and *Hantaviridae* [20–25]. During our surveillance, no positive results were obtained in the black and brown rats for the five viral taxa tested. DNA virus screening was directed towards poxviruses, as human cases of cowpox virus were reported with an origin in infected pet rats [30]. Nonetheless, poxviruses were rarely reported in rodents. This is in line with our results, where no poxviruses have been identified in brown and black rats, and also with other previous surveys in Europe [31,32]. In other rodent species, orthopoxviruses have been detected (including the brown rat, gerbil, common vole, and Western Mediterranean mouse [20,21,27]).

A meta-analysis on the prevalence of orthohantaviruses in rodents (~5%; [33] highlighted that their global prevalence is a point of concern. The role of rodents as reservoirs prompted our analysis. There is no molecular evidence of hantavirus in brown and black rats from São Tomé and Príncipe. Its prevalence in rodents worldwide is variable, ranging from 0% to almost 30% (e.g. [27,32,34,35]). To what concerns the African continent, the orthohantavirus Anjo strain was found in the black rat in Madagascar [36].

Flaviviruses, which encompass virus such as the yellow fever virus, have been detected in rodents from Senegal, Uganda and Kenya [24,37], including black rats. Current global changes, such as increased trading and traveling, have altered the distribution, transmission cycles and dynamics of flaviviruses, highlighting the critical need for ongoing surveillance studies. Our survey did not detect any flavivirus in black or brown rats, consistent with other studies [38]. On the contrary, antibodies reactive with flaviviruses have been found in black rats and house mice in Mexico [39]. In fact, for all viral taxa included, our results could not determine if the tested animals had been exposed to the targeted viruses, but rather if they were currently infected. For example, in Europe, a seroprevalence of 3.6-11.7% against LCMV (arenavirus) was found [40–42], while for Puumala virus and Dobrava virus (hantaviruses) it stays below

0.5% [40]. Thus, serological testing combined with molecular testing could have provided more information regarding the zoonotic risk associated with these species.

While no coronavirus RNA has been found in our dataset, their remarkable capacity to overcome species barriers and adapt to hosts close to humans emphasizes the critical need for comprehensive research on coronavirus infections [e.g. 43]. Other efforts to survey coronavirus presence in rodents have yielded conflicting results. While in Europe and the neighbor Gabon no coronaviruses have been found in rodent species [31,32], in the Republic of Congo two animals were found to be positive for coronavirus RNA (the Congo forest mouse, *Deomys ferrugineus*, and the big-eared swamp rat, *Malacomys longipes*). Moreover, the ability of SARS-CoV-2 variants of concern to spread has been demonstrated [44] along with evidence of urban rats´ exposure to the virus, due to the presence of anti-SARS-CoV-2 antibodies detection. Collectively, these results underscore the critical need to monitor potential reservoir species in insular environments to effectively control pathogen spread.

Our results were negative for the presence of arenaviruses consistent with the findings of a study conducted in Italy [32]. However, this group of viruses is known for causing persistent, silent infections in rodents and severe even lethal human diseases [45]. Lassa virus is endemic in West Africa and black rats were shown to be competent reservoirs [46]. Its prevalence among black rats is especially high in some regions of Nigeria, reaching >75% prevalence [22]. While Lassa fever cases have not yet been reported in São Tomé and Príncipe, viral presence in neighboring countries, such as Nigeria, Benin, and Togo, increases the risk of arrival to the islands. Interestingly, in West African countries, where the invasive black rat competes with the native Natal multimammate mouse (*Mastomys natalensis*) and primary reservoir host of Lassa virus, the zoonotic spillover risk is lower when in the presence of the black rat [47]. Moreover, a significant diversity of mammarenaviruses was discovered within a limited host sample size and a relatively small geographical area, reinforcing that Angola is a hotspot for arenaviruses. These viruses have been identified in species such as *Mastomys natalensis*,

Micaelamys namaquensis, and *Mus triton*, which were not included in the current study but are in close contact with other rodent species and humans [48].

In our survey, we tested representative samples (liver, spleen, and feces) of all individuals that covered the broad tissue and cell tropism of the selected viruses. Samples were collected at different locations, seasons, and years, which ensured unbiased sampling of males and females, adults and juveniles. The detection techniques were appropriate and of high quality, with negative and positive controls confidently confirming their applicability. Yet, positive controls corresponded to samples with active infections and high viral loads, and the samples from this study were collected in apparently healthy animals, with no conspicuous clinical signs that could indicate disease and an ongoing active infection. Hence, target viruses might be present but in very low viral loads that may not have generated viremia and go unnoticed in PCR. Some of these viruses do not cause lifelong persistent infections, and sampling may not coincide with the space-time presence of infected hosts, reducing the likelihood of viral detection. Several other limitations might explain the results obtained. Indeed, the low number of samples analyzed from only a few sites and covering short periods of time might have precluded the detection of active and acute infections. Further surveys should aim at a larger sample size to increase the likelihood of detection. Other limitations include sample viability (despite their storage in RNA later, they were not tested on-site), low sensitivity of the PCR or the limited specificity of the primers employed, notwithstanding their pan/universal application, considering that the targeted viruses might have a particular evolution in this insular region. To overcome these limitations, more sensitive methodologies might be considered as methods of choice for future surveillance studies, such as NGS and digital PCR. Indeed, NGS allows unbiased detection of all microorganisms in a sample and has been successfully employed to identify uncommon and novel infectious viral agents [reviewed in 49], while digital PCR is a highly sensitive and precise molecular technique that detects minimal amounts of target even in the presence of a high number of non-target templates [50]. The combination of surveillance

and correct management of invasive species is expected to result in significant improvements in both public and animal health and the conservation of biodiversity.

These apparently negative results should be taken with caution, as they do not exclude a potential infection state among *R. rattus* and *R. norvegicus* from São Tomé and Príncipe, being advisable that their handling should be safely conducted due to the extensively documented risk they pose as hosts for zoonotic viruses. The publication of absence data on rat-borne pathogens provides a more complete and unbiased assessment of zoonotic risk. Even if these are true negative results, they remain valuable for constructing distribution models and are essential to identifying potential areas at risk.

Here, we show the results of a molecular survey investigating zoonotic viruses in invasive rodent species. Our study highlighted an absence of such viruses among rats from São Tomé and Príncipe, making, to our knowledge, the first survey of its kind conducted in this insular country. Rodents are widely distributed in urban, peri-urban, and rural environments, where their increasing proximity of mammals to humans and livestock, amplifies the vulnerability of native species, in line with a One Health approach, we propose maintaining long-term surveillance of zoonotic viruses in São Tomé and Príncipe. This surveillance effort should be complemented with serological studies and extended to include other viral zoonoses of concern (e.g., rabies, Ebola virus, and highly pathogenic avian influenza) and other species. Furthermore, viruses with limited zoonotic potential but known to infect humans – such as rotavirus, norovirus, astrovirus, and kobuvirus – should also be investigated, particularly those sporadically detected in wild rats and mice.

Acknowledgments

Positive control samples were kindly provided by Dr. Susana Lopes, CIBIO/UP (*Coronaviridae*: SARS-CoV-2); Dr. João Queirós, CIBIO/UP (*Flaviviridae*: Bagaza virus); Department of Biomedicine, University of Basel, as participant of the European Virus Archive

(*Arenaviridae*, Lymphocytic choriomeningitis virus: pLCMV_Pol-I-L-ARM); Biomedical Research Center of the SLovak Academy of Sciences, as participant of the European Virus Archive (*Hantaviridae*, Hantaan virus: HTNV 78-118).

We want to acknowledge the Direção das Florestas e da Biodiversidade (DFB) of São Tomé and Príncipe for approving the collection of samples by local legislation, and the Centro de Investigação Agronómica e Tecnológica de São Tomé e Príncipe (CIAT-STP) for certifying the shipment of samples.

References

1. Melo, M.F.P.P. de Bird Speciation in the Gulf of Guinea. PhD Thesis. *Institute of Evolutionary Biology, University of Edinburgh, UK.* **2006**.
2. Ceriáco, L.M.P.; Marques, M.P.; Jacquet, F.; Nicolas, V.; Colyn, M.; Denys, C.; Sardinha, P.C.; Bastos-Silveira, C. Description of a New Endemic Species of Shrew (Mammalia, Soricomorpha) from Príncipe Island (Gulf of Guinea). *Mammalia* **2015**, *79*, 325–341, doi:10.1515/mammalia-2014-0056.
3. Dutton, J. Introduced Mammals in São Tomé and Principe: Possible Threats to Biodiversity. *Biodivers Conserv* 1994, *3*, 927–938.
4. Ganjeer, T.; Patyal, A.; Shakya, S.; Parkar, S.S.; Shukla, A.; Chandrakar, C.; Naik, V. Rodent Borne Zoonoses: A Brief Review. *Pharma Innov* **2021**, *10*, 721–725, doi:10.22271/tpi.2021.v10.i8Sk.7406.
5. Lowe, S.; Browne, M.; Boudjelas, S.; De Poorter, M. *100 of the World's Worst Invasive Alien Species: A Selection from the Global Invasive Species Database*; Invasive Species Specialist Group Auckland, 2000; Vol. 12;.
6. Drake, D.R.; Hunt, T.L. Invasive Rodents on Islands: Integrating Historical and Contemporary Ecology. *Biol Invasions* **2009**, *11*, 1483–1487, doi:10.1007/s10530-008-9392-1.
7. Harris, D.B. Review of Negative Effects of Introduced Rodents on Small Mammals on Islands. *Biol Invasions* **2009**, *11*, 1611–1630, doi:10.1007/s10530-008-9393-0.

8. Russell, J.C.; Meyer, J.Y.; Holmes, N.D.; Pagad, S. Invasive Alien Species on Islands: Impacts, Distribution, Interactions and Management. *Environ Conserv* **2017**, *44*, 359–370, doi:10.1017/S0376892917000297.
9. Battersby, S.A. Rodents as Carriers of Disease. In *Rodent pests and their control*; CABI: UK, 2015; pp. 81–100.
10. Himsworth, C.G.; Parsons, K.L.; Jardine, C.; Patrick, D.M. Rats, Cities, People, and Pathogens: A Systematic Review and Narrative Synthesis of Literature Regarding the Ecology of Rat-Associated Zoonoses in Urban Centers. *Vector-Borne and Zoonotic Diseases* **2013**, *13*, 349–359, doi:10.1089/vbz.2012.1195.
11. Chen, R.-X.; Gong, H.-Y.; Wang, X.; Sun, M.-H.; Ji, Y.-F.; Tan, S.-M.; Chen, J.-M.; Shao, J.-W.; Liao, M. Zoonotic Hantaviridae with Global Public Health Significance. *Viruses* **2023**, *15*, 1705, doi:10.3390/v15081705.
12. Capizzi, D.; Bertolino, S.; Mortelliti, A. Rating the Rat: Global Patterns and Research Priorities in Impacts and Management of Rodent Pests. *Mamm Rev* **2014**, *44*, 148–162, doi:10.1111/mam.12019.
13. Purdy, M.A.; Harrison, T.J.; Jameel, S.; Meng, X.-J.; Okamoto, H.; Van der Poel, W.H.M.; Smith, D.B. ICTV Virus Taxonomy Profile: Hepeviridae. *Journal of General Virology* **2017**, *98*, 2645–2646, doi:10.1099/jgv.0.000940.
14. Radoshitzky, S.R.; Buchmeier, M.J.; Charrel, R.N.; Gonzalez, J.-P.J.; Günther, S.; Hepojoki, J.; Kuhn, J.H.; Lukashevich, I.S.; Romanowski, V.; Salvato, M.S.; et al. ICTV Virus Taxonomy Profile: Arenaviridae 2023. *Journal of General Virology* **2023**, *104*, doi:10.1099/jgv.0.001891.
15. Simmonds, P.; Becher, P.; Bukh, J.; Gould, E.A.; Meyers, G.; Monath, T.; Muerhoff, S.; Pletnev, A.; Rico-Hesse, R.; Smith, D.B.; et al. ICTV Virus Taxonomy Profile: Flaviviridae. *Journal of General Virology* **2017**, *98*, 2–3, doi:10.1099/jgv.0.000672.
16. Woo, P.C.Y.; De Groot, R.J.; Haagmans, B.; Lau, S.K.P.; Neuman, B.W.; Perlman, S.; Sola, I.; Van Der Hoek, L.; Wong, A.C.P.; Yeh, S.H. ICTV Virus Taxonomy Profile:

Coronaviridae 2023. *Journal of General Virology* **2023**, *104*, doi:10.1099/jgv.0.001843.
17. McInnes, C.J.; Damon, I.K.; Smith, G.L.; McFadden, G.; Isaacs, S.N.; Roper, R.L.; Evans, D.H.; Damaso, C.R.; Carulei, O.; Wise, L.M.; et al. ICTV Virus Taxonomy Profile: Poxviridae 2023. *Journal of General Virology* **2023**, *104*, doi:10.1099/jgv.0.001849.
18. Akhtar, N.; Hayee, S.; Idnan, M.; Nawaz, F.; BiBi, S. Rodents Human Zoonotic Pathogens Transmission: Historical Background and Future Prospects. In *Rodents and Their Role in Ecology, Medicine and Agriculture*; IntechOpen, 2023.
19. Crump, J.A.; Murdoch, D.R.; Baker, M.G. Emerging Infectious Diseases in an Island Ecosystem: The New Zealand Perspective. *Emerg Infect Dis* **2001**, *7*, 767–772, doi:10.3201/eid0705.017501.
20. Lourie, B.; Nakano, J.H.; Kemp, G.E.; Setzer, H.W. Isolation of Poxvirus from an African Rodent. *Journal of Infectious Diseases* **1975**, *132*, 677–681, doi:10.1093/infdis/132.6.677.
21. Tantawi, H.H.; Zaghloul, T.M.; Zakaria, M. Poxvirus Infection in a Rat (Rattus Norvegicus) in Kuwait. *Int J Zoonoses* **1983**, *10*, 28–32.
22. Happi, A.N.; Olumade, T.J.; Ogunsanya, O.A.; Sijuwola, A.E.; Ogunleye, S.C.; Oguzie, J.U.; Nwofoke, C.; Ugwu, C.A.; Okoro, S.J.; Otuh, P.I.; et al. Increased Prevalence of Lassa Fever Virus-Positive Rodents and Diversity of Infected Species Found during Human Lassa Fever Epidemics in Nigeria. *Microbiol Spectr* **2022**, *10*, doi:10.1128/spectrum.00366-22.
23. Kumakamba, C.; Niama, F.R.; Muyembe, F.; Mombouli, J.-V.; Kingebeni, P.M.; Nina, R.A.; Lukusa, I.N.; Bounga, G.; N'Kawa, F.; Nkoua, C.G.; et al. Coronavirus Surveillance in Wildlife from Two Congo Basin Countries Detects RNA of Multiple Species Circulating in Bats and Rodents. *PLoS One* **2021**, *16*, e0236971, doi:10.1371/journal.pone.0236971.

24. Diagne, M.M.; Ndione, M.H.D.; Di Paola, N.; Fall, G.; Bedekelabou, A.P.; Sembène, P.M.; Faye, O.; De Andrade Zanotto, P.M.; Sall, A.A. Usutu Virus Isolated from Rodents in Senegal. *Viruses* **2019**, *11*, doi:10.3390/v11020181.
25. Klempa, B.; Fichet-Calvet, E.; Lecompte, E.; Auste, B.; Aniskin, V.; Meisel, H.; Denys, C.; Koivogui, L.; ter Meulen, J.; Krüger, D.H. Hantavirus in African Wood Mouse, Guinea. *Emerg Infect Dis* **2006**, *12*, 838.
26. Li, Y.; Meyer, H.; Zhao, H.; Damon, I.K. GC Content-Based Pan-Pox Universal PCR Assays for Poxvirus Detection. *J Clin Microbiol* **2010**, *48*, 268–276, doi:10.1128/JCM.01697-09.
27. Herrero-Cófreces, S.; Mougeot, F.; Sironen, T.; Meyer, H.; Rodríguez-Pastor, R.; Luque-Larena, J.J. Viral Zoonoses in Small Wild Mammals and Detection of Hantavirus, Spain. *Emerg Infect Dis* **2022**, *28*, doi:10.3201/eid2806.212508.
28. Escutenaire, S.; Mohamed, N.; Isaksson, M.; Thorén, P.; Klingeborn, B.; Belák, S.; Berg, M.; Blomberg, J. SYBR Green Real-Time Reverse Transcription-Polymerase Chain Reaction Assay for the Generic Detection of Coronaviruses. *Arch Virol* **2007**, *152*, 41–58, doi:10.1007/s00705-006-0840-x.
29. Vina-Rodriguez, A.; Sachse, K.; Ziegler, U.; Chaintoutis, S.C.; Keller, M.; Groschup, M.H.; Eiden, M. A Novel Pan- Flavivirus Detection and Identification Assay Based on RT-QPCR and Microarray. *Biomed Res Int* **2017**, *2017*, doi:10.1155/2017/4248756.
30. Ninove, L.; Domart, Y.; Vervel, C.; Voinot, C.; Salez, N.; Raoult, D.; Meyer, H.; Capek, I.; Zandotti, C.; Charrel, R.N. Cowpox Virus Transmission from Pet Rats to Humans, France. *Emerg Infect Dis* **2009**, *15*, 781–784, doi:10.3201/eid1505.090235.
31. de Cock, M.P.; de Vries, A.; Fonville, M.; Esser, H.J.; Mehl, C.; Ulrich, R.G.; Joeres, M.; Hoffmann, D.; Eisenberg, T.; Schmidt, K.; et al. Increased Rat-Borne Zoonotic Disease Hazard in Greener Urban Areas. *Science of The Total Environment* **2023**, *896*, 165069, doi:10.1016/j.scitotenv.2023.165069.

32. Di Bartolo, I.; De Sabato, L.; Ianiro, G.; Vaccari, G.; Dini, F.M.; Ostanello, F.; Monini, M. Exploring the Potential of Muridae as Sentinels for Human and Zoonotic Viruses. *Viruses* **2024**, *16*, 1041, doi:10.3390/v16071041.
33. Obando-Rico, C.J.; Valencia-Grajales, Y.F.; Bonilla-Aldana, D.K. Prevalence of Orthohantavirus in Rodents: A Systematic Review and Meta-Analysis. *Travel Med Infect Dis* **2023**, *51*, 102504, doi:10.1016/j.tmaid.2022.102504.
34. Hönig, V.; Kamiš, J.; Maršíková, A.; Matějková, T.; Stopka, P.; Mácová, A.; Růžek, D.; Kvičerová, J. Orthohantaviruses in Reservoir and Atypical Hosts in the Czech Republic: Spillover Infection and Indication of Virus-Specific Tissue Tropism. *Microbiol Spectr* **2022**, *10*, doi:10.1128/spectrum.01306-22.
35. Lukman, N.; Kosasih, H.; Ibrahim, I.N.; Pradana, A.A.; Neal, A.; Karyana, M. A Review of Hantavirus Research in Indonesia: Prevalence in Humans and Rodents, and the Discovery of Serang Virus. *Viruses* **2019**, *11*, 698, doi:10.3390/v11080698.
36. Kikuchi, F.; Senoo, K.; Arai, S.; Tsuchiya, K.; Sơn, N.; Motokawa, M.; Ranorosoa, M.; Bawm, S.; Lin, K.; Suzuki, H.; et al. Rodent-Borne Orthohantaviruses in Vietnam, Madagascar and Japan. *Viruses* **2021**, *13*, 1343, doi:10.3390/v13071343.
37. Wang, D.; Yang, X.; Ren, Z.; Hu, B.; Zhao, H.; Yang, K.; Shi, P.; Zhang, Z.; Feng, Q.; Nawenja, C.V.; et al. Substantial Viral Diversity in Bats and Rodents from East Africa: Insights into Evolution, Recombination, and Cocirculation. *Microbiome* **2024**, *12*, 72, doi:10.1186/s40168-024-01782-4.
38. Sotomayor-Bonilla, J.; García-Suárez, O.; Cigarroa-Toledo, N.; Cetina-Trejo, R.C.; Espinosa-García, A.C.; Sarmiento-Silva, R.E.; Machain-Williams, C.; Santiago-Alarcón, D.; Mazari-Hiriart, M.; Suzán, G. Survey of Mosquito-Borne Flaviviruses in the Cuitzmala River Basin, Mexico: Do They Circulate in Rodents and Bats? *Trop Med Health* **2018**, *46*, 35, doi:10.1186/s41182-018-0117-6.
39. Cigarroa-Toledo, N.; Talavera-Aguilar, L.G.; Baak-Baak, C.M.; García-Rejón, J.E.; Hernandez-Betancourt, S.; Blitvich, B.J.; Machain-Williams, C. Serologic Evidence of

Flavivirus Infections in Peridomestic Rodents in Merida, Mexico. *J Wildl Dis* **2016**, *52*, 168–172, doi:10.7589/2015-05-116.

40. Kallio-Kokko, H.; Laakkonen, J.; Rizzoli, A.; Tagliapietra, V.; Cattadori, I.; Perkins, S.E.; Hudson, P.J.; Cristofolini, A.; Versini, W.; Vapalahti, O.; et al. Hantavirus and Arenavirus Antibody Prevalence in Rodents and Humans in Trentino, Northern Italy. *Epidemiol Infect* **2006**, *134*, 830–836, doi:10.1017/S0950268805005431.
41. Lledó, L.; Gegúndez, M.I.; Saz, J.V.; Bahamontes, N.; Beltrán, M. Lymphocytic Choriomeningitis Virus Infection in a Province of Spain: Analysis of Sera from the General Population and Wild Rodents. *J Med Virol* **2003**, *70*, 273–275, doi:10.1002/jmv.10389.
42. Tagliapietra, V.; Rosà, R.; Hauffe, H.C.; Laakkonen, J.; Voutilainen, L.; Vapalahti, O.; Vaheri, A.; Henttonen, H.; Rizzoli, A. Spatial and Temporal Dynamics of Lymphocytic Choriomeningitis Virus in Wild Rodents, Northern Italy. *Emerg Infect Dis* **2009**, *15*, 1019–1025, doi:10.3201/eid1507.081524.
43. Menachery, V.D.; Graham, R.L.; Baric, R.S. Jumping Species—a Mechanism for Coronavirus Persistence and Survival. *Curr Opin Virol* **2017**, *23*, 1–7, doi:10.1016/j.coviro.2017.01.002.
44. Montagutelli, X.; Prot, M.; Levillayer, L.; Salazar, E.B.; Jouvion, G.; Conquet, L.; Beretta, M.; Donati, F.; Albert, M.; Gambaro, F.; et al. Variants with the N501Y Mutation Extend SARS-CoV-2 Host Range to Mice, with Contact Transmission 2021.
45. Moolla, N.; Weyer, J. The Arenaviridae. In *Emerging and Reemerging Viral Pathogens*; Elsevier, 2020; pp. 69–100.
46. Agbonlahor, D.E.; Erah, A.; Agba, I.M.; Oviasogie, F.E.; Ehiaghe, A.F.; Wankasi, M.; Eremwanarue, O.A.; Ehiaghe, I.J.; Ogbu, E.C.; Iyen, R.I.; et al. Prevalence of Lassa Virus among Rodents Trapped in Three South-South States of Nigeria. *J Vector Borne Dis* **2017**, *54*, 146–150.
47. Eskew, E.A.; Bird, B.H.; Ghersi, B.M.; Bangura, J.; Basinski, A.J.; Amara, E.; Bah, M.A.; Kanu, M.C.; Kanu, O.T.; Lavalie, E.G.; et al. Reservoir Displacement by an

Invasive Rodent Reduces Lassa Virus Zoonotic Spillover Risk. *Nat Commun* **2024**, *15*, 3589, doi:10.1038/s41467-024-47991-1.

48. Těšíková, J.; Krásová, J.; Goüy de Bellocq, J. Multiple Mammarenaviruses Circulating in Angolan Rodents. *Viruses* **2021**, *13*, 982, doi:10.3390/v13060982.

49. Bassi, C.; Guerriero, P.; Pierantoni, M.; Callegari, E.; Sabbioni, S. Novel Virus Identification through Metagenomics: A Systematic Review. *Life* **2022**, *12*, 2048, doi:10.3390/life12122048.

50. Nazir, S. Medical Diagnostic Value of Digital PCR (DPCR): A Systematic Review. *Biomedical Engineering Advances* **2023**, *6*, 100092, doi:10.1016/j.bea.2023.100092.
