# Supplementary material for: Viral zoonoses assessment in invasive rodent species from São Tomé and Príncipe
Source: PLoS One. 2026 Feb 2;21(2):e0341657. doi: 10.1371/journal.pone.0341657 (PMC12863471; doi:10.1371/journal.pone.0341657)
Supplement: Attachment — Submitted filename: Response to Reviewers.pdf [file pone.0341657.s004.pdf]

Reviewer #1: The article Viral zoonoses assessment in invasive rodent species from Sao Tomé and Principe is well-written; however, it does not constitute a significant contribution to the topic of viral zoonoses carried by two invasive rat species. While the subject is interesting—addressing invasive rat species on an island, where literature has reported their highest risk to native wildlife—the authors also note that these rats have been present in the area for a long time and, in fact, only coexist with two species of wild mammals (two shrews). From my perspective, the article is not suitable for publication in this journal, which seeks higher-impact articles, at least not in its current form. Below, I provide a series of suggestions to consider for improving the text.

We are grateful for the reviewer's constructive feedback and fully acknowledge the concerns expressed. The present study used samples originally collected as part of a separate project investigating the dietary preferences of species inhabiting São Tomé and Príncipe. As outlined in the manuscript, the study's limitations reflect the logistical challenges of the field sample collection, but are also due to financial constraints. Despite these limitations, we consider it valuable to report negative results to reduce knowledge gaps and avoid redundant research efforts.

The sample size used for the analyses (86 specimens) is very small, and although the authors themselves discuss this limitation, it would be desirable to obtain a larger sample, particularly for the brown rat (R. norvegicus). Even if this species is not very abundant on the island, the current sample (15 or 14 specimens) remains insufficient. This may be due to the limited number of traps employed, prompting my recommendation to increase their use in future efforts.

Sampling was designed to cover a gradient of human activity that would reflect a range of rodent-human contact intensities and aimed at an average of 20 samples per species per habitat. The sampling design aimed to balance the effort across land-use types, i.e., forest, cacao, palm, and village, rather than balance the number of samples per species. While black rats (*R. rattus*) are abundant in the four sampled habitats and appear well-represented, brown rats (*R. norvegicus*) are rare outside human settlements and are represented by fewer samples. Thus, the variation in sample size among and between species reflects species differences in habitat use and abundance. Regarding the sampling effort, we used Tomahawk-like traps baited with oat cookies and peanut butter and placed 20 meters apart in a 1-hectare plot, and sampling lasted 4 days. These settings are in agreement with those used in other studies of these species.

As mentioned in the manuscript, we conducted two field trips, one pilot trip in December 2021 to test for several trapping techniques, and a second field trip from May to September 2022 across 12 localities evenly spread among the different habitats (cocoa, palm, forest and villages), where the most successful trapping techniques were employed. While not mentioned in the manuscript, no *R. norvegicus* were successfully

trapped in the pilot field trip, hinting for their difficult sampling, which was further confirmed in the second field trip. However, as we were able to collect a few individuals in the second field trip, we opted to include them in the analyses for a more complete overview of the zoonotic potential of rodents in São Tomé and Príncipe.

In the Materials and Methods section, it is not specified that sampling followed a gradient, an aspect that is mentioned later in the text.

This information was included in the Materials and Methods section: "These habitats represent a gradient of human activity, with sampling reflecting varying degrees of contact intensity between rats and humans."

Although the Materials and Methods section states that specimens were weighed, measured, and sexed, these data are not discussed further. It would be useful to include this information at least in a table to provide an overview of the collected population's condition. Additionally, it is mentioned that specimens were collected along an anthropogenic gradient, but the number of specimens per land cover type is not specified. I suggest summarizing this information in a table as well. Even though no specimens tested positive, data on their distribution across the landscape would enrich the text.

As suggested, a new table summarizing the information on morphological parameters (mean ± S.D.) of male and female samples of black rats and brown rats collected per habitat was added to the manuscript (c.f. Table 3). The values for the morphological parameters are within the range of those reported for these species (www.gbif.org). Moreover, a table with individual data on those parameters was also included as supplementary information (c.f. S1 Table).

I recommend conducting additional tests, possibly for antibody presence, which, given the current results, might provide further insights into the presence of these viruses in rat populations.

We fully agree with the reviewer's recommendation; however, the current project's financial framework preclude the inclusion of additional tests in the current study.

Finally, I suggest enhancing the text with a map of the study area to provide context for international readers unfamiliar with the region.

A map of the study area was included in the manuscript (Figure 1).

I have left additional comments within the text.

All additional comments within the text have been carefully addressed, including corrections to formatting and clarification of sample differences noted between the Materials and Methods and Results and Discussion sections.

Reviewer #2: Despite having negative results, the research is well-structured in terms of results and discussions. However, it is necessary to explain how the sample size was determined and why the N varies from one species to another.

Sampling was designed to cover a gradient of human activity that would reflect a range of rodent-human contact intensities, and aimed at collecting an average of 20 samples per species per habitat. The sampling design intended to balance the effort across land-use types, i.e., forest, cacao, palm, and village, rather than balance the number of samples per species. Thus, since black rats (*R. rattus*) are abundant in the four sampled habitats, they appear well-represented. In contrast, brown rats (*R. norvegicus*) are rare outside human settlements and are represented by fewer samples. The variation in sample size among and between species reflects differences in habitat use and abundance.
